# Improving localization accuracy for non-invasive automated early left ventricular origin localization approach

**DOI:** 10.3389/fphys.2023.1183280

**Published:** 2023-06-26

**Authors:** Shijie Zhou, Raymond Wang, Avery Seagren, Noah Emmert, James W. Warren, Paul J. MacInnis, Amir AbdelWahab, John L. Sapp

**Affiliations:** ^1^ The Department of Chemical, Paper and Biomedical Engineering, Miami University, Oxford, OH, United States; ^2^ The Department of Computer Science and Software Engineering, Miami University, Oxford, OH, United States; ^3^ Mason High School, Mason, OH, United States; ^4^ The Department of Physiology and Biophysics, Dalhousie University, Halifax, NS, Canada; ^5^ Cardiology Division, Department of Medicine, Queen Elizabeth II Health Sciences Centre, Halifax, NS, Canada

**Keywords:** k-nearest neighbors (KNN) algorithm, ventricular tachycardia, pace-mapping, radiofrequency ablation, ECG

## Abstract

**Background:** We previously developed a non-invasive approach to localize the site of early left ventricular activation origin in real time using 12-lead ECG, and to project the predicted site onto a generic LV endocardial surface using the smallest angle between two vectors algorithm (SA).

**Objectives:** To improve the localization accuracy of the non-invasive approach by utilizing the K-nearest neighbors algorithm (KNN) to reduce projection errors.

**Methods:** Two datasets were used. Dataset #1 had 1012 LV endocardial pacing sites with known coordinates on the generic LV surface and corresponding ECGs, while dataset #2 included 25 clinically-identified VT exit sites and corresponding ECGs. The non-invasive approach used “population” regression coefficients to predict the target coordinates of a pacing site or VT exit site from the initial 120-m QRS integrals of the pacing site/VT ECG. The predicted site coordinates were then projected onto the generic LV surface using either the KNN or SA projection algorithm.

**Results:** The non-invasive approach using the KNN had a significantly lower mean localization error than the SA in both dataset #1 (9.4 vs. 12.5 mm, *p* < 0.05) and dataset #2 (7.2 vs. 9.5 mm, *p* < 0.05). The bootstrap method with 1,000 trials confirmed that using KNN had significantly higher predictive accuracy than using the SA in the bootstrap assessment with the left-out sample (*p* < 0.05).

**Conclusion:** The KNN significantly reduces the projection error and improves the localization accuracy of the non-invasive approach, which shows promise as a tool to identify the site of origin of ventricular arrhythmia in non-invasive clinical modalities.

## Introduction

Sudden cardiac arrest is a major cause of death in developed countries, with approximately 350,000 deaths per year in the United States alone (Al-Khatib et al., 2017). The majority of those events are caused by ventricular arrhythmias (VAs) (Tang et al., 2017). Catheter ablation has emerged as an established therapeutic option for the treatment of VAs (Reddy et al., 2007; Kuck et al., 2010; Sapp et al., 2016). Accurate identification of the substrate responsible for the VA is key to the success of the modality and may be facilitated using 12-lead ECG to non-invasively localize the breakthrough site from which a focal ventricular tachycardia (VT) or premature ventricular contraction (PVC) arises, from an exit pathway of a transmural re-entry, or from which a re-entrant circuit exits the central isthmus to activate the “normal” myocardium. Computer-aided methods for rapidly and automatically localizing the site of early ventricular activation origin in real-time using 12-lead ECG can be particularly helpful for catheter ablation of VA (Cronin et al., 2020). Several non-invasive algorithms based on the 12-lead ECG have been proposed for localizing the site of early ventricular activation origin with varying degrees of accuracy (Josephson et al., 1981; Miller et al., 1988; Betensky et al., 2011; Yokokawa et al., 2012; Efimova et al., 2015; Misra et al., 2018; Alawad and Wang, 2019; He et al., 2020). Recent studies using deep learning—based VT exit/PVC origin localization have shown moderate localization accuracy (Yang et al., 2018; Gyawali et al., 2020), and the “black box” nature of these deep learning models makes it difficult to interpret how input variables interact to identify the VT exit/PVC origin site (Zhou et al., 2021).

Locating the site of origin within 10 mm is of great clinical significance. We have previously developed a non-invasive automated approach that combines information from 12-lead ECG recordings and a generic LV endocardial mesh surface consisting of 238 area elements to localize the site of early left-ventricular (LV) activation origin (Sapp et al., 2017; Zhou et al., 2019). We have shown that, using the non-invasive automated approach based on the 12-lead ECG (Sapp et al., 2017; Zhou et al., 2019), spatial localization of the site of early LV activation origin can be achieved without significant loss of accuracy in comparison with our 120-lead Electrocardiographic Imaging (ECGI) (Zhou et al., 2018a; Zhou et al., 2018b); ECGI uses CT/MRI volumes to delineate the cardiac anatomy and determine the relative location of body surface electrodes to reconstruct epicardial electrical events by a mathematical process known as calculation of inverse solution (Zhou et al., 2018a; Zhou et al., 2018b). The non-invasive automatic approach demonstrated a mean localization error of 12.2 ± 8.14 mm (Sapp et al., 2017; Zhou et al., 2019). Briefly, the non-invasive automated approach was based on the hypothesis of a linear relationship between coordinates of early left ventricular activation sites and ECG lead potentials (QRS integrals); a dataset comprising coordinates (exported from an electroanatomic mapping (EAM) system) of 1012 LV endocardial pacing sites and their corresponding ECGs was used to calculate population-derived regression coefficients; the non-invasive automated approach uses these population-derived regression coefficients to predict the target coordinates of a pacing site or VA from the initial 120-m QRS integrals of the pacing site ECG or the VA ECG. The predicted pacing-site/VA origin site coordinates are projected onto one of the 238 triangular area elements of the generic LV endocardial mesh surface using the smallest angle between two vectors algorithm (named SA algorithm), so that the projected site can be targeted for ablation. However, the prolate ellipsoid geometry of the normal LV shape, with a ratio of 2:1 from echocardiographic long axis dimension to minor axis dimension (Udelson, 2017), may lead to projection errors when using the SA algorithm.

In this study, we hypothesized that K-Nearest Neighbors (KNN) algorithm can reduce the projection error and improve the localization performance of the non-invasive automated approach. The paper is organized as follows, Section 2 describes the methods in detail, Section 3 presents the results, and Section 4 concludes the study and discusses its limitations and future research.

## Methods

### Clinical datasets

This study utilized two datasets (Sapp et al., 2017; Zhou et al., 2019; Zhou et al., 2020). The first dataset (#1) comprised 1012 LV endocardial pacing sites pooled from 38 patients, which were exported from an EAM system (Carto 3, Biosense Webster, Inc., Irvine, CA, United States) and included corresponding ECGs (Sapp et al., 2017; Zhou et al., 2019). The second dataset (#2) included 25 clinically-identified LV endocardial VT exit sites with corresponding ECGs (Zhou et al., 2020). All participating patients gave written informed consent; the study protocol was approved by the Institutional Research Ethics Board (Nova Scotia Health Authority, Halifax, Canada).

### Datasets description

In a previous study, we constructed a generic LV endocardial mesh surface consisting of 238 triangles, derived from the necropsy specimen of a normal human heart (Hren et al., 1998). The average distance between the centers of the 238 triangles was found to be 5.4 ± 1.4 mm (mean ± SD). In the current study, the Cartesian coordinates of each pacing site or clinically-identified VT exit site were manually registered from the patient-specific EAM geometry onto one of the 238-triangle centers of the generic LV endocardial surface by two independent observers (Drs Sapp and AbdelWahab) (Sapp et al., 2017; Zhou et al., 2019; Zhou et al., 2020). The registration process had an interobserver variability of approximately 3.7 mm in the surrounding area (Zhou et al., 2020). For each pacing site/clinical-identified VT exit site, the QRS integral was calculated over the initial 120 m of the QRS complex for the 8 independent leads (I, II, V1-V6) of the 12-lead ECG (∫QRS, in microvolt-seconds) (Sapp et al., 2017; Zhou et al., 2018a; Zhou et al., 2018b; Zhou et al., 2019; Zhou et al., 2020) by using summing up the area of the 120 m QRS window.

### The non-invasive automated approach

The non-invasive automated approach based on a training set (*n* samples) with a multiple linear regression (MLR) model can be applied to the 238-triangle generic LV endocardial mesh surface, provided the 8-variable set (*P*_*:i*_, *i* = 1, . . ., *k* = 8) can be generated from the 8 independent leads ECG (I, II, V1-V6) for the training-set pacing sites with known coordinates *x*_*j*_, *y*_*j*_, *z*_*j*_, (*j* = 1, . . . *n*.) (Sapp et al., 2017; Zhou et al., 2019). The MLR model with intercept is used to determine “population” regression coefficients in the regression equations linking each of the 3 coordinates of a known pacing site *x*_*j*_, *y*_*j*_, *z*_*j*_, with the values of the corresponding QRS integrals, *P*_*ji*_, where the subscript *i* indicates one of the 8 leads:

$$\begin{array}{ccc} x_{j}=\alpha_{0}+\sum_{i=1}^{8}\alpha_{i}P_{ji} & y_{j}=\beta_{0}+\sum_{i=1}^{8}\beta_{i}P_{ji} & z_{j}=\gamma_{0}+\sum_{i=1}^{8}\gamma_{i}P_{ji} \end{array}$$ (1)

Here $\alpha_{i}$ , $\beta_{i}$ , and $\gamma_{i}$ (*i* = 0, . . ., 8) are calculated “population” regression coefficients based on the training set (*n* samples). The least-squared solution to this problem yielded the estimates of the “population” regression coefficients. Next, the calculated 3 sets of the “population” regression coefficients are used in MLR models mapping the coordinates of an unknown pacing-site origin or an unknown clinically-induced VT (coordinates $\hat{x}$ , $\hat{y}$ , $\hat{z}$ ) with the values of the 8 QRS integrals (*V*_*i*_) of the pacing site ECG or the clinically-induced VT ECG

$$\begin{array}{ccc} \hat{x}=\alpha_{0}+\sum_{i=1}^{8}\alpha_{i}V_{i} & \hat{y}=\beta_{0}+\sum_{i=1}^{8}\beta_{i}V_{i} & \hat{z}=\gamma_{0}+\sum_{i=1}^{8}\gamma_{i}V_{i} \end{array}$$ (2)

The unknowns in this set of equations are the coordinates of the pacing-site origin or the clinically-induced VT exit site, $\hat{x}$ , $\hat{y}$ , $\hat{z}$ . Once the coordinates of the pacing-site origin or the induced VT exit site are calculated, they are projected onto one of the 238-triangle centers of the generic LV endocardial mesh surface as the targeted site.

### Projection algorithm based on the smallest angle between two vectors (SA algorithm)

To project the predicted pacing/VT exit site $\hat{x}$ , $\hat{y}$ , $\hat{z}$ onto one of the 238-triangle centers of the generic LV endocardial mesh surface, we have previously used vector multiplication to determine the smallest angle between two vectors. In the 238-triangle generic LV endocardial mesh surface, there exist 238 vectors ( $A_{t}$ , *t* = 1, … 238), each extending from the center of the generic LV cavity to a corresponding triangle centroid. A vector B is obtained from the center of the generic LV cavity to the predicted pacing/VT exit site ( $\hat{x}$ , $\hat{y}$ , $\hat{z}$ ). To compute all angles between $A_{t}\;\left(t=1,\ldots ,238\right)$ vectors and the B vectors, we utilize the following formulated:

$$\theta_{t}=\cos^{-1}\left(\frac{A_{t}∙B}{\left|A_{t}\right|∙\left|B\right|}\right);t=1,\ldots ,238$$ (3)

After calculating Eq. 3, the smallest angle, $\theta_{smallest}$ , can be obtained from one of the 238 $\theta_{t}\;\left(t=1,\ldots ,238\right)$ . The center of the corresponding triangle, t, serves as the final projected site on the generic LV endocardial mesh surface.

### Projection algorithm based on K-Nearest neighbors (KNN) algorithm

**FIGURE 1:**
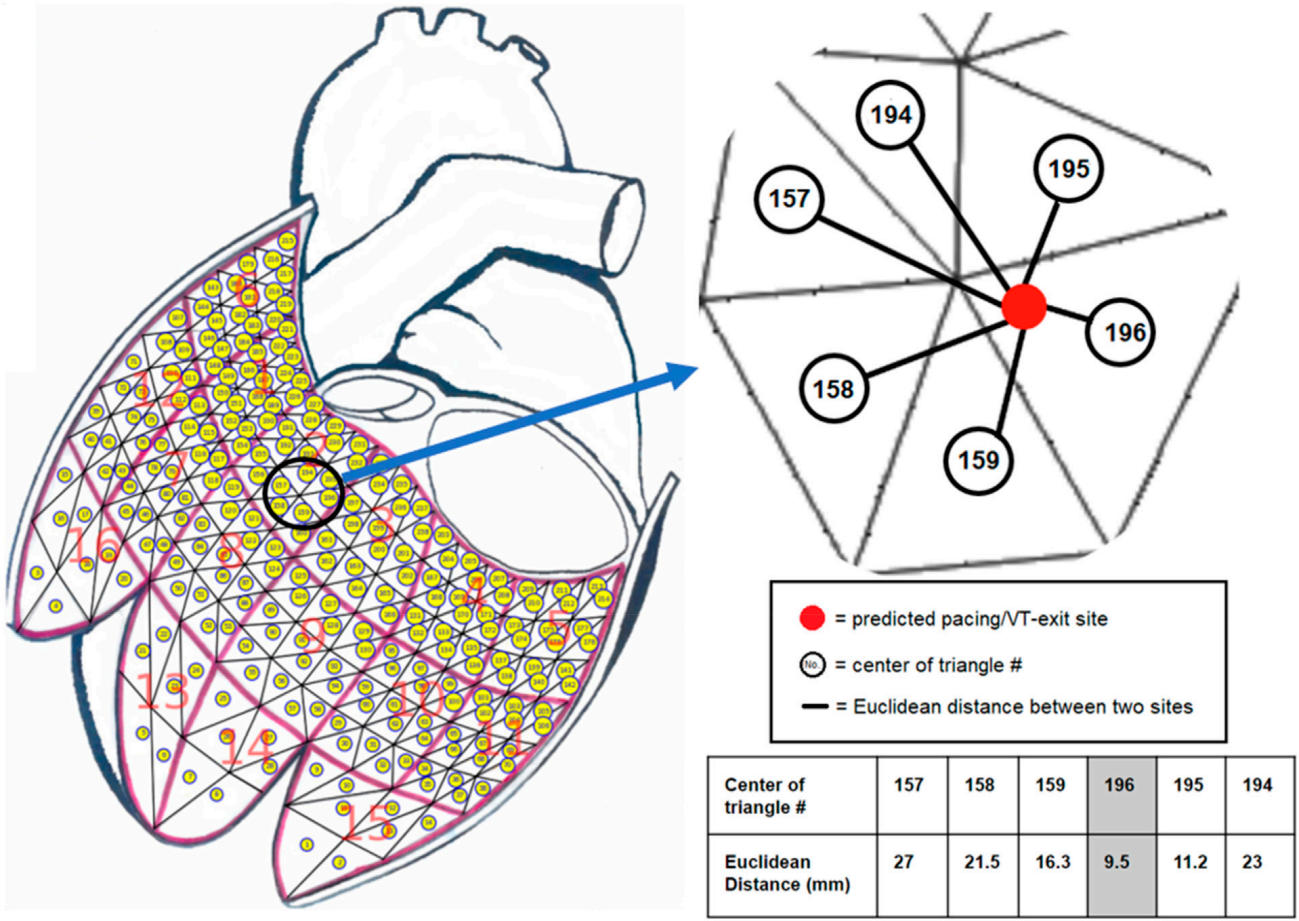
The K-nearest neighbors (KNN) algorithm with Euclidean distance measurement was used to project a predicted pacing/VT-exit site marked by the red ball onto one of the 238-triangle centers of the generic LV endocardial mesh surface. The example illustrates that a predicted pacing/VT-exit site marked by the red ball was projected onto the No. 196 triangle center by finding the shortest distance from all of the 238 Euclidean distances calculated by the predicted pacing/VT-exit site and all of the 238-triangle centers of the generic LV endocardial mesh surface.

The K-nearest neighbors (KNN) algorithm is a non-parametric approach for classification and regression tasks in supervised learning (Bzdok et al., 2018). In this study, the KNN is used to predict the class of the test data by calculating the distance between the test data and all the training points, then to select the ‘K’ number of points which is close to the test data. In this study, the test data is the predicted pacing/VT exit site ( $\hat{x}$ , $\hat{y}$ , $\hat{z}$ ); the training points are all of the 238-triangle centers of the generic LV endocardial mesh surface; ‘K’ was assigned to 1 which means that the object is simply assigned to the class of its nearest neighbor. In other words, the predicted pacing/VT exit site ( $\hat{x}$ , $\hat{y}$ , $\hat{z}$ ) is classified into a class; and there are 238 classes obtained from the 238-triangle centers. Then, the distances between the predicted pacing/VT exit site and all other 238-triangle centers were calculated for finding the nearest neighbors by ranking sites by increasing distance. Figure 1 illustrates the nearest neighbors of the predicted pacing/VT exit site that are closest in dataspace. The predicted pacing/VT exit site ( $\hat{x}$ , $\hat{y}$ , $\hat{z}$ ) is then assigned to the No. 196 triangle center based on the votes from the KNN, and is projected onto the 196th triangle of the LV endocardial mesh surface as the final targeted site.

### Localization accuracy assessment based on the two datasets

#### Dataset #1

To evaluate the localization performance of the non-invasive automated approach, the entire dataset #1 (*n* = 1,012) was randomly partitioned into a training set with 80% of the entire set (*n* = 810) and a test set with the remaining 20% (*n* = 202). The “population” regression coefficients were calculated from the training set (*n* = 810) using Eq. 1, and then applied as constants with the ECG variables extracted from the 8 independent leads ECG of any activation sequence of interest initiated at the unknown site obtained from the test set (*n* = 202)., The resulting coordinates $\hat{x}$ , $\hat{y}$ , $\hat{z}$ of the unknown pacing site can be calculated using Eq. 2 with the known “population” regression coefficients and the ECG variables for the unknown pacing site of interest. Finally, the predicted pacing site was projected onto the generic LV endocardial mesh surface (triangle centroid ( $x_{e},y_{e},z_{e}$ )) using the two projection algorithms, resulting in an estimated pacing site origin on one of the 238 triangles of the generic LV mesh surface.

In addition, the bootstrap method with replacement was used to assess the statistical inference (Efron and Tibshirani, 1993). To estimate the optimistic bias of the proposed projection algorithm, the mean error with standard deviation was calculated and applied to the left-out sample (*n* = 1,012/e≃371), which served as a test set. The mean and standard deviation of 1,000 bootstrap trials was calculated. The standard error was used to construct the 95%-confidence interval of the localization performance. The Minitab’s boxplot was used to visualize the distribution of data and identify any potential outliers. To determine the outliers, the Minitab involved identifying data points that are more than 1.5 times the interquartile range (IQR) below the first quartile or above the third quartile.

The actual pacing site is known and specified on the 238-triangle generic LV endocardial mesh surface as triangle centroid ( $x_{r},y_{r},z_{r}$ ), the accuracy of pacing-site localization can be then assessed from the geodesic distance between ( $x_{e},y_{e},z_{e}$ ) and ( $x_{r},y_{r},z_{r}$ ), measured on the curved surface of the generic LV, both located on the surface of the generic LV. The localization accuracy was then estimated from the geodesic distance (in mm) approximated as an arc length on the sphere with center at the center-point of the generic LV.

#### Dataset #2

We evaluated the localization performance of the non-invasive automated approach using the KNN projection algorithm on dataset #2, which consists of 25 clinically-identified VT-exit sites. We calculated the “population” regression coefficients using the entire dataset #1 (*n* = 1,012), and compared the results to those of the non-invasive automated approach using the SA projection algorithm. In the Dataset #2, we used the Euclidean distance to assess the localization accuracy of the non-invasive automated approach using the SA projection algorithm for predicting the 25 clinically-identified VT-exit sites in our previous study (Zhou et al., 2020). To ensure consistency in this study, the localization error of the VT exit site was assessed by measuring the Euclidean distance between the clinically-identified site and the estimated site both on the generic LV endocardial mesh surface.

## Results

### Localization performance assessment based on the dataset #1

#### Localization performance of the non-invasive automated approach using the two projection algorithms based on a test set (*n* = 202)

**FIGURE 2:**
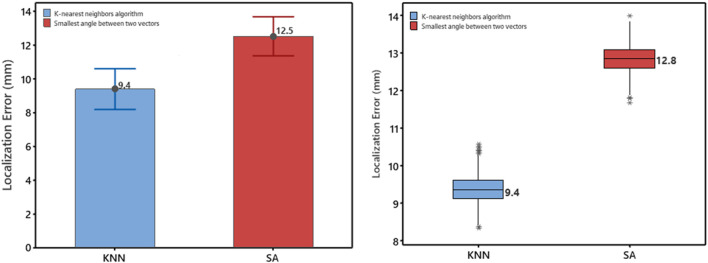
Left Panel, Comparison based on the test set (*n* = 202) for the error measured as geodesic distance using the two projection algorithms (KNN, SA). Mean values are shown numerically. Right Panel: Box plot of localization error for using the two projection algorithms (KNN, SA). Plots represent data for mean localization error in terms of geodesic distance on the generic LV-endocardial surface for the left-out sample (*n* = 1,012/e≃371). Boxes represent interquartile ranges; a line inside the box marks the median, “whiskers” above and below the box indicate range, ∗∗ represent outliers.

The study evaluated the localization error of a non-invasive automated approach using two projection algorithms. Geodesic distance was calculated from the centroid of the predicted triangle to the centroid of the known reference triangle on the 238-triangle generic LV endocardial mesh surface. After deriving “population” regression coefficients from a training set (*n* = 810), the accuracy of the approach was assessed on a test set (*n* = 202). The mean accuracy of the non-invasive automated approach was 9.4 mm and 12.5 mm for using the KNN projection and the SA projection algorithms, respectively. Figure 2 (left panel) shows that the mean localization error using the KNN projection algorithm was significantly lower than that of the SA projection algorithm (9.4 vs. 12.5 mm, *p* < 0.05).

#### Localization performance of the non-invasive automated approach using the two projection algorithms based on bootstrap assessment

**FIGURE 3:**
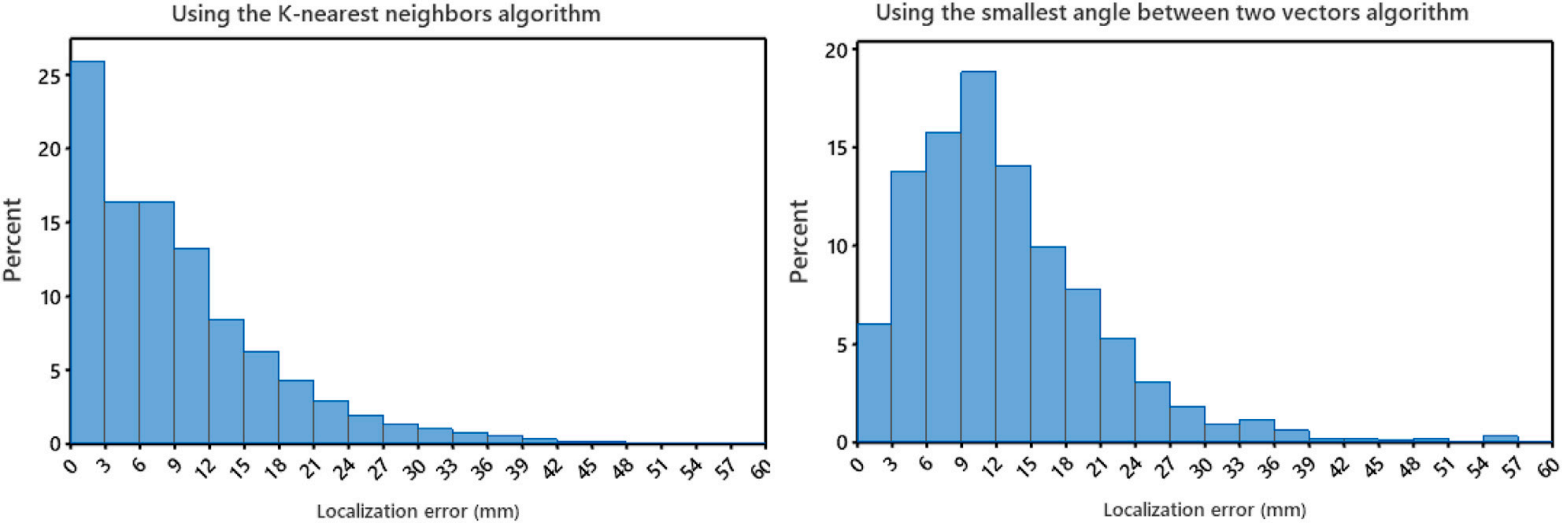
Left panel: the distributions for the non-invasive automated approach using the K-nearest neighbors (KNN) projection algorithm of the bootstrap left-out-sample errors, based on localization errors on the LV endocardial surface for 1,000 bootstrap trials. Right panel: the distributions for the non-invasive automated approach using the smallest angle between two vectors algorithm of the bootstrap left-out-sample errors, based on localization errors on the LV endocardial surface for 1,000 bootstrap trials. Error was measured as geodesic distance (approximated by the arc length) between the centroid of the projected triangle and the centroid of the pacing-site triangle on the 238-triangle generic LV endocardial mesh surface.

The localization performance of the non-invasive automated approach using the two projection algorithms was further evaluated in terms of geodesic distance between the centroid of the projected and actual pacing site, measured on the generic LV endocardial mesh surface. The bootstrap method with replacement, using 1,000 trials (Efron and Tibshirani, 1993), was employed to summarize the results. Table 1 and in Figure 2 (right panel) provide an overview of the results. As shown in Figure 2 (right panel), the KNN projection algorithm yielded a significantly lower mean localization errors compared to the SA projection algorithm (KNN vs SA, *p* < 0.05) in the left-out sample (n≃371). The distributions of the left-out-sample errors are depicted in Figure 3, based on the pacing-site localization error measured as a geodesic distance in the generic LV endocardial mesh surface, for the 1,000 bootstrap trials using the non-invasive automated approach with the two projection algorithms.

**TABLE 1:** Bootstrap method with replacement (Efron and Tibshirani, 1993), using 1,000 trials, was used; the left-out sample had *n* = 1,012/e≃371 pacing sites. Three g-variables quantify accuracy of localization by eight-predictor regression in terms of geodesic distance (in mm) from the centroid of the predicted triangle to the centroid of the pacing-site triangle on the 238-triangle generic LV endocardial mesh surface; gmean, mean value; gsd, standard deviation; gmedian, median value; Pctl, percentile.

Algorithms	Variable	Mean	SD	Median	5th pctl	95th pctl
Using the K-nearest-neighbors	gmean	9.4	0.4	9.4	8.7	10.1
	gsd	8.5	0.4	8.5	7.7	9.3
	gmedian	7.2	0.4	7.3	6.6	8.1
Using the smallest angle	gmean	12.8	0.4	12.8	12.1	13.6
	gsd	8.7	0.5	8.7	7.6	9.7
	gmedian	11.2	0.4	11.2	10.4	12.0

### Localization performance assessment based on the dataset #2

**FIGURE 4:**
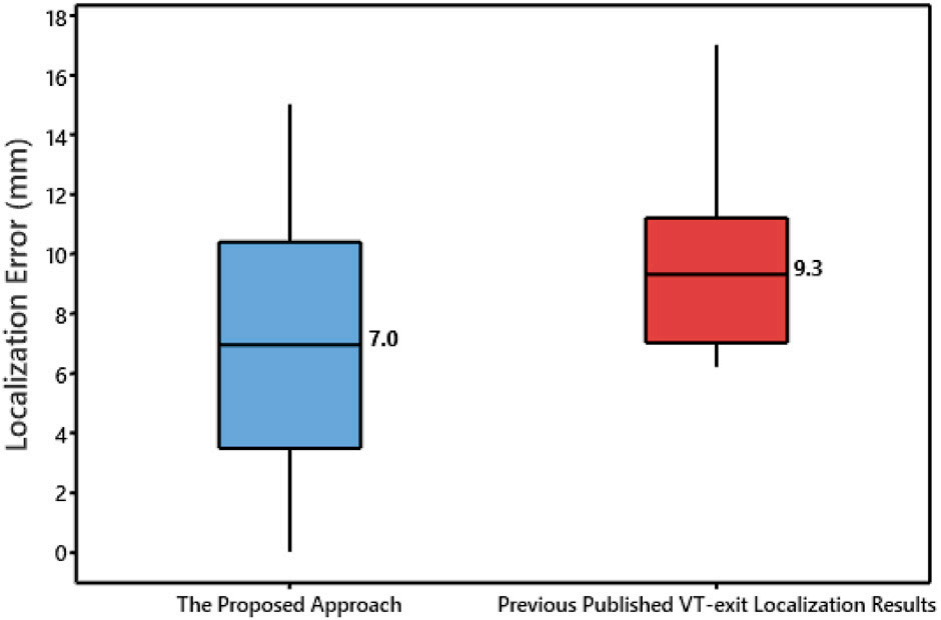
Box plot of localization error of the 25 VT exit sites for using the non-invasive automated approach based the two projection algorithms. Plots represent data for mean localization error in terms of Euclidean distance.

**FIGURE 5:**
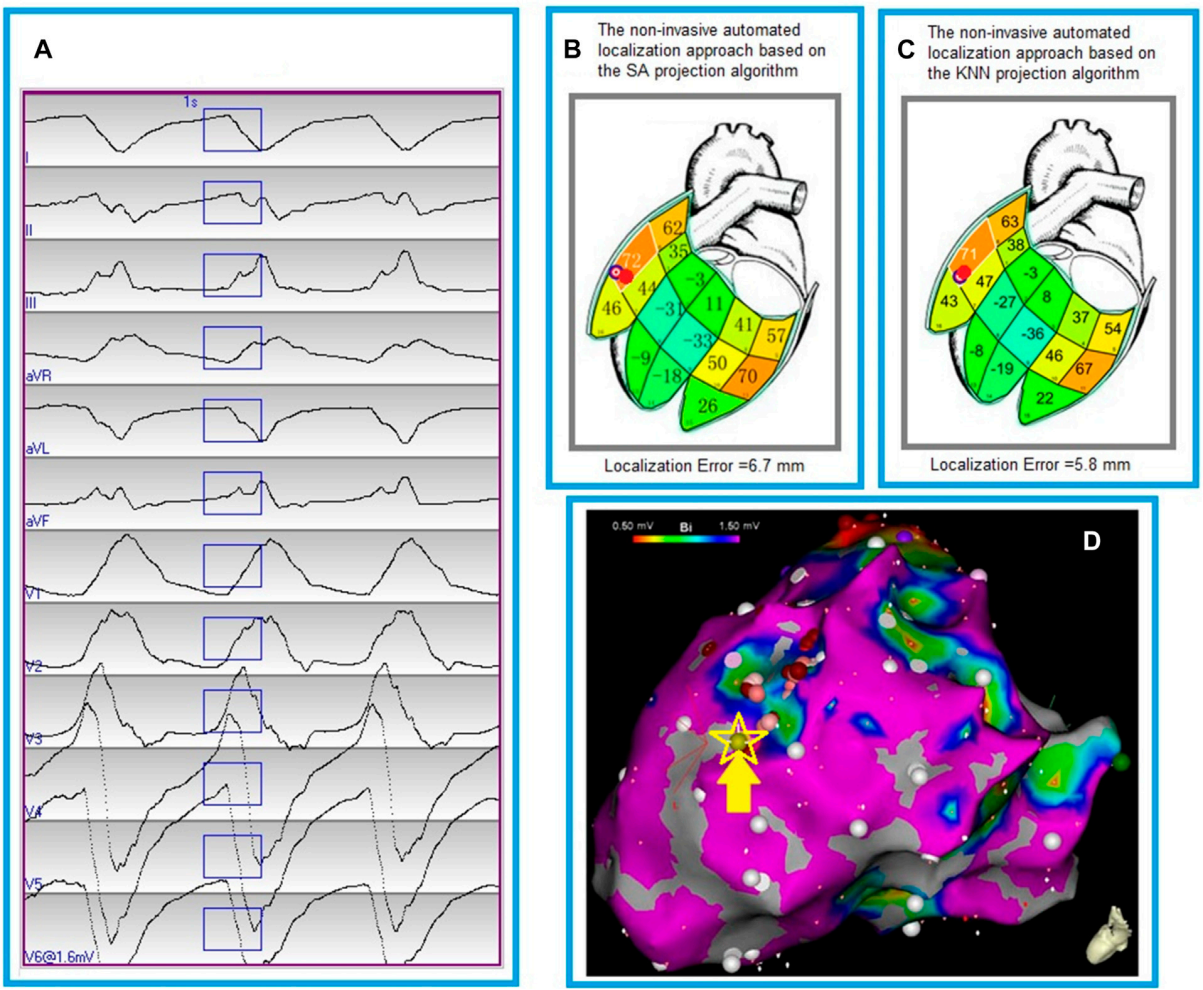
Localization of a ventricular tachycardia (VT) exit by the non-invasive automated localization using the two projection algorithms. **(A)**, The recorded 12-lead ECG of an induced monomorphic VT during the procedure. The onset of one VT beat was automatically detected (Kemmelings et al., 1994); the user can edit the onset of the 120 m window (rectangle box) if correction is necessary. **(B)**, Bull’s eye icon that indicates the estimated VT-exit locations using the non-invasive automated approach based on the smallest angle between two vectors (SA algorithm). The red ball indicates the VT reference site on the 238-triangle generic LV endocardial mesh surface, which was registered manually from an endocardial electroanatomic mapping map (panel D). Localization error of the VT exit site is 6.7 mm between the bull’s eye icon and the red ball. The large number within each segment is the correlation coefficient (%) for match by the 12-lead ECG VT pattern with population based 12-lead ECG templates; the small number identifies the segment. **(C)**, Bull’s eye icon that indicates the estimated VT-exit locations using the non-invasive automated approach based on the K-nearest neighbors algorithm (KNN). The red ball registered manually from an endocardial electroanatomic mapping map (panel D) indicates the VT reference site on the 238-triangle generic LV endocardial mesh surface. Localization error of the VT exit site is 5.8 mm between the bull’s eye icon and the red ball. The large number within each segment is the correlation coefficient (%) for match by the 12-lead ECG VT pattern with population based 12-lead ECG templates; the small number identifies the segment. **(D)**, an endocardial electroanatomic substrate map, with areas featuring bipolar signal amplitude ≥1.50 mV in purple, and the site of VT exit (identified by contact mapping) depicted by the yellow arrow, yellow starand gold ball.

Figure 4 shows the localization errors of 25 clinically-identified VT exit sites in the dataset #2, based on the non-invasive automated approach using the two different projection algorithms. The mean localization error of the non-invasive automated approach, using the KNN projection algorithm (named proposed approach), was significantly lower than that of the non-invasive automated approach using the SA projection algorithm (published in reference 23). (7.2 vs. 9.5 mm, *p* < 0.05). Figure 5 illustrates the non-invasive automated approach for localizing the exit site of a ventricular tachycardia (VT) using two projection algorithms. This VT had a cycle length of 315 m, with right bundle branch block-type morphology in lead V1, and a rightward axis (Figure 5A). The site of exit was identified at the mid-apical anterolateral wall inferior to the anterolateral papillary muscle. The VT exit site was localized to the more apical portion of the mid-anterolateral segment identified by the non-invasive automated approach using the two projection algorithms, respectively (Figure 5B for using the SA projection algorithm; Figure 5C for using the KNN projection algorithm). The electroanatomic substrate map is shown in Figure 5D, with the site of VT exit identified (yellow arrow, yellow star and gold ball).

## Discussion

In this study, we introduced the K-nearest neighbors (KNN) algorithm to improve the localization performance of a non-invasive automated approach by reducing the projection error. The KNN algorithm was used to project the predicted pacing/VT-exit site onto a generic LV endocardial mesh surface, and compare its accuracy to the smallest angle between two vectors projection algorithm. The non-invasive automated approach utilizing the KNN projection algorithm achieved a mean localization accuracy of < 10 mm in both datasets, highlighting its clinical significance.

Based on the comprehensive assessments, we conclude that the KNN projection algorithm can enhance the localization accuracy of the non-invasive automated approach in clinical cardiac electrophysiology. The KNN algorithm does not rely on any machine learning model that requires a pre-existing training on a dataset to make predictions. In other words, the KNN does not require any training, which saves the training dataset and uses it only when making real-time predictions to learn. This makes the KNN algorithm much faster than other training-based algorithms, such as, random forest or support vector machine. In addition, the KNN requires knowing the number of categories (one or more), which means that the K value has a powerful effect on the KNN performance. In our specific situation, the ‘K’ value was required to be 1, which completely solved the most prominent issue—the optimal K number for determining the KNN performance. Therefore, the 1-NN (‘K’ NN) algorithm was directly used to calculate the Euclidean distances between the predicted pacing/VT-exit site and all of the 238-triangle centers of the generic LV endocardial mesh surface, finding the shortest distance for the calculated 238 Euclidean distances.

Our study represents a significant step towards improving the localization accuracy of the non-invasive automated approach for real-time localization of early LV activation origin, which has potential applications for catheter ablation of VA (Cronin et al., 2020) and targeting VT locations for substrate modification using cardiac stereotactic body radiotherapy (cSBRT) (Qian et al., 2021). Recently cardiac SBRT as a non-invasive alternative has been shown to provide a viable option for VT which is refractory to ablation and medication (Cuculich et al., 2017). While ECGI has been proposed as a means to identify VT substrate for guiding cardiac SBRT (Graham et al., 2019; Samson et al., 2020), its spatial accuracy is limited and depends on several factors (Hohmann et al., 2019). Septal activation, for example, is inherently difficult to represent on the epicardial surface (Duchateau et al., 2019). Duchateau et al. compared ECGI with invasive epicardial mapping, and found an inadequate correlation between the two modalities, with an average distance of 75 mm from the invasively mapped focal breakthrough locations to the predicted origin sites (Bhaskaran et al., 2019). Our proposed approach provides a promising foundation for future studies in the non-invasive cSBRT. However, there are several limitations of this study. Specifically, the proposed study has limited applicability in the right ventricle (RV) endocardium and epicardium. To overcome this limitation, future studies could utilize the same method to identify the site of early RV/epicardial activation origin. Additionally, the proposed study is based on a generic LV endocardial mesh surface and does not account for the patient-specific LV endocardial surface. Future studies would explore a non-invasive ECG-image-based mapping approach that relies on personalized ventricular surfaces from CT/MRI scans and the proposed approach for identifying the site of early ventricular activation origin in both ventricles.

## Conclusion

The K-nearest neighbors (KNN) algorithm can greatly reduce the projection error, improve the localization accuracy of the non-invasive automated approach. By utilizing the KNN projection algorithm, the non-invasive automated approach outperforms any prior published approached, which may potentially facilitate the use of non-invasive clinical modalities in identifying the site of origin of ventricular arrhythmia.

## Data Availability

The original contributions presented in the study are included in the article/supplementary materials, further inquiries can be directed to the corresponding author.
